# Pharmacokinetic Characteristics of a Single Cannabidiol Dose in Oil and Treat Forms and Health Impacts After 30 Days of Administration in Dogs

**DOI:** 10.3390/ani15101470

**Published:** 2025-05-19

**Authors:** Phattharakan Kamutchat, Sasithorn Limsuwan, Nattaya Leewichit, Natthasit Tansakul

**Affiliations:** 1 Graduate Program in Animal Health and Biomedical Sciences, Faculty of Veterinary Medicine, Kasetsart University, Bangkok 10900, Thailand; phattharakan.kam@ku.th (P.K.); nattaya.le@ku.th (N.L.); 2 Institute of Food Research and Product Development, Kasetsart University, Bangkok 10900, Thailand; sasithorn.limsu@ku.th; 3 Department of Pharmacology, Faculty of Veterinary Medicine, Kasetsart University, Bangkok 10900, Thailand

**Keywords:** cannabidiol, pharmacokinetics, dogs, hemp, oil formulation, treats, safety

## Abstract

Cannabidiol (CBD) has garnered significant interest in veterinary therapeutics, yet the pharmacokinetic and safety profiles of its various formulations remain incompletely characterized. This study compared the pharmacokinetics (PK) and health effects of CBD administered as oil (OG, 5 mg/kg) and treats (TG, 50 mg) in 16 healthy mixed-breed dogs over 30 days. Plasma CBD concentrations were measured using liquid chromatography–tandem mass spectrometry (LC-MS/MS), and the PK parameters were analyzed using non-compartmental methods. The CBD-infused rice bran oil formulation (OG) achieved a significantly higher dose-normalized maximum plasma concentration (C_max_, 58.40 vs. 21.29 kg·ng/mL/mg) and area under the curve (AUC_0-inf_, 305.85 vs. 141.75 h·kg·ng/mL/mg) compared to the treats (TG). The treat formulation exhibited relative reductions in bioavailability, with AUC and Cmax values approximately 2.2- and 2.7-fold lower than the oil group. The terminal half-life (~9.66 h OG vs. 8.52 h TG) and time to peak (2.38 h OG vs. 3.63 h TG) did not differ significantly. CBD accumulation occurred with repeated dosing but declined rapidly post-cessation. The hematological and biochemical analyses revealed no clinically adverse effects, though minor erythrocyte and eosinophil fluctuations were noted. The oil formulation demonstrated superior absorption, while both forms were well-tolerated. These findings highlight the impact of formulation on CBD absorption and support further research into optimized delivery methods for veterinary applications.

## Simple Summary

This study evaluated the comparative plasma pharmacokinetics of two oral CBD formulations—oil-based (5 mg/kg) and treat-mixed (50 mg)—in dogs following a single-dose administration and 30 days of repeated dosing. The serial blood analyses quantified the CBD concentrations and assessed the blood test parameters. The key findings demonstrated that the oil formulation achieved significantly higher bioavailability, with greater peak plasma concentrations and systemic exposure compared to the treat form. Both formulations were well-tolerated, with no clinically adverse effects observed, though transient alterations in certain blood profiles were noted. CBD exhibited accumulation during the study but rapid clearance upon discontinuation. These results suggest that oil-based CBD may optimize therapeutic consistency due to enhanced absorption, while treats provide a practical alternative. This study provides empirical evidence to guide veterinarians and pet owners in selecting formulation-specific dosing strategies.

## 1. Introduction

Cannabidiol (CBD) is one of over 120 phytocannabinoids found in cannabis plants, primarily hemp. In recent years, CBD has gained significant attention for its potential health benefits in humans and animals [1]. The therapeutic effects of CBD are currently being studied across a range of health conditions due to its interaction with the body’s endocannabinoid system, a network of receptors involved in the regulation of various processes [2,3,4]. Although more research is needed to fully understand the effects of CBD in animals, anecdotal evidence and preliminary studies indicate that it is frequently taken into consideration for a number of medical conditions, including anti-inflammatory, pain-relieving, and anxiety-reducing effects, as well as anti-seizure properties [3,5,6,7]. While research is ongoing, many veterinarians and pet owners are investigating the possibilities of utilizing CBD to treat a variety of health concerns in their pets [8,9].

Despite CBD demonstrating significant therapeutic potential in animal studies, its pharmacokinetics (PK) profiles in companion animals, particularly canines, remain inadequately characterized. CBD can be administered to dogs through various routes and it has been studied in a variety of dose forms in animal trials [10] These include oil-based liquid, capsules, soft chewable formulations [11,12,13,14], microencapsulated oil beads and transdermal cream [15], intranasal preparations, and suppositories [16], both water-soluble and nanoemulsion forms [14]. The pharmacokinetics of CBD in dogs are characterized by low oral bioavailability, moderate distribution, and are mostly eliminated via the fecal route [5,17,18].

Following oral administration, the half-life of CBD in canines ranges from 4 to 10 h, contingent upon the formulation and dosage [5,12,14,15,17]. This relatively short half-life indicates that frequent dosing may be necessary to maintain therapeutic concentrations, particularly for chronic conditions such as persistent pain or epilepsy. CBD-infused oil is one of the most commonly used formulations in veterinary therapy, often administered orally, and is widely researched for its application [9,10,11]. CBD treats, on the other hand, have gained prominence as a convenient method of administering CBD to canines. These treats are formulated to be palatable and easily incorporated into a dog’s daily regimen. However, there is limited empirical evidence regarding its pharmacokinetics and impact on a dog’s health over time.

CBD administration in dogs has been shown to affect various blood parameters, particularly the liver enzymes. Multiple studies have reported an increase in alkaline phosphatase (ALP) activity in dogs receiving CBD treatment [9,19,20]. This elevation in ALP was observed even when CBD was administered at different dosages and durations [9,19]. Interestingly, while the ALP levels increased, other liver function parameters and most hematological and serum chemistry variables remained within normal reference ranges [9,20].

The efficacy of pharmaceutical agents may vary depending on the route of administration, formulation, and dosage. The pharmacokinetic characteristics of various dosage forms in target animal species have not been thoroughly examined. It is essential to conduct further studies, considering the diverse factors that influence plasma CBD concentrations [14]. The primary objective of the present investigation was to compare the plasma behavior profiles of CBD following oral administration once daily in two dosage forms: CBD-infused in vegetative oil and CBD-mixed in a snack as a treat. Additionally, the health effects following sub-chronic ingestion of both forms were evaluated.

Understanding CBD pharmacokinetics and its health impact in dogs is crucial for establishing the proper dosages, potential side effects, and interactions with other medications. This current study of cannabidiol’s pharmacokinetics and health effects in dogs over a 30-day period may provide valuable insights into its use and safety in canine medicine. This research could inform veterinary practices, potentially leading to more effective and safer cannabidiol-based treatments for dogs. Moreover, the comparison between oil and treat forms of administration may help to determine the most efficient delivery method, which could influence future product development and dosing recommendations in veterinary care.

## 2. Materials and Methods

### 2.1. Chemical and CBD Preparation

The CBD standard was obtained from Cerliliant^®^ (Round Rock, TX, USA). CBD-D3 was produced by Cambridge Isotope Laboratories, Inc. (Tewksbury, MA, USA). Certified CBD powder was sourced from Salus Bioceutical (Bangkok, Thailand) Co., Ltd., with a purity level exceeding 99%, as confirmed by a certified third-party testing laboratory. HPLC and LC/MS grades of acetonitrile and methanol were obtained from Labscan Co., Ltd. (Bangkok, Thailand). A CBD-infused oil 5% formulation (OG group) was prepared by dissolving CBD powder in refined rice bran oil with high natural antioxidants, including oryzanol, phytosterols, and vitamin E. The CBD preparation and assay were modified following a previously published protocol [14].

The GMP dog food company produced CBD treats (TG group) on a small scale for research purposes. The primary components included corn, rice bran, rice bran oil, and water. After mixing, the resulting mixture was expected to yield CBD at a concentration of 50 mg per treat. Subsequently, all treat samples underwent a 30 min heating process at 100 °C. To quantify the CBD content in the treats via HPLC, testing was performed on 30 samples of 300 finished treat products. The results revealed that the CBD levels in all samples matched the expected concentration, with a standard deviation of less than 2.3%. All CBD preparations were stored in tightly sealed packaging in dark containers and placed in the control temperature room (under 25 °C) until used for animal consumption within a week.

### 2.2. Animals

A parallel design was used to randomly assign 16 mixed-breed healthy dogs, aged 2.5–5.5 years and weighing 12.6–19.4 kg with an ideal body score condition, equally to two treatment groups. As reported in a prior study by Limsuwan et al. (2024) [14], the sample size was determined using G*Power software, version 3.1.9.4, with the parameters set at α = 0.05, two-tailed, and a power of 0.8. This calculation indicated that a sample size of *n* = 8 per group is sufficient to identify the differences in Cmax between two independent groups in a pilot study, aligning well with the ethical guidelines [21]. Before treatment, the dogs were allowed at least 14 days to acclimatize after not receiving any medication for a month. During their stay, they were housed separately in kennels and cared for according to the university’s procedures. As part of the acclimatization process, physical examinations, clinical observations, hematology tests, and blood chemistry tests were conducted. A single meal was provided to each dog daily as per the dog’s individual metabolizable energy requirement. The animals were fasted overnight before being treated. In the weeks preceding and following treatment, clinical signs and adverse events (e.g., vomit, diarrhea, lethargy, drowsiness, drooling, urinary incontinence) were monitored twice a day, from 8:00 to 9:00 and from 15:00 to 16:00.

### 2.3. Experimental and Dosing Design

Each overnight-fasted animal received a single oral dose of CBD in the oil-based group (OG) based on its actual body weight (BW), with a target dosage of 5 mg/kg BW in individually adjusted dosing volumes. The administration was conducted by placing the dose into the buccal cavity using a syringe. In the CBD-treat group (TG), each serving contained 50 mg of CBD that was administered as one piece of the treat to dogs for self-ingestion. The animal was observed closely to ensure complete consumption of the treat.

### 2.4. Specimens and Collection

At the following intervals, blood samples were drawn and preserved in a tube containing Potassium EDTA using a no. 22” IV catheter through cephalic or saphenous venipuncture: after a single oral ingestion of both forms: −1 day, 30 min, and then 1, 2, 3, 6, 10, 24, 30 and 48 h. To observe the plasma CBD concentration after the PK study, the dogs were administered oral doses for 30 consecutive days, and additional blood samples were taken 3 h after dosing on days 1, 2, 16, 30, and 31. As soon as the samples were collected, they were placed on ice and shielded from light until centrifugation. Following a 15 min centrifugation at 3000× *g* at 4 °C, the plasma and serum were collected into aliquots that were labeled and coded by the laboratory. In addition, blood samples were taken on days 0–15–30 for hematology and blood chemistry analyses, which were placed in a tube with potassium EDTA and serum clot activators. All aliquots were shipped in an icepack box and placed in a dark cover box to freeze at −80 °C until the analysis could be completed within 80 days.

### 2.5. Quantitative Measurement of Plasma-Containing CBD

Quantitative analysis was conducted using an Agilent Technologies 1260 Infinity system, which includes an autosampler, binary pump, and thermostated column compartment, coupled with an Agilent Technologies 6460 Triple Quadrupole mass spectrometer (Agilent, Waldbronn, Germany). The gradient elution in chromatography was achieved on a Thermo Scientific Accucore C18 column (2.1 mm × 50 mm, 2.6 μm; P/N: 17626-052130) with a guard column of the same phase. A modified LC-MS/MS in-house protocol, adapted from the guideline for bioanalytical method validation and previous research [22,23], was employed for the quantitative analysis of CBD in canine plasma specimens. The mobile phase A comprised 0.1% formic acid in water, while mobile phase B consisted of 0.1% formic acid in acetonitrile. The elution protocol for analyte separation involved increasing mobile phase B from 60% to 95% at 6.00 min. The column was subsequently re-equilibrated with 60% B for 4.00 min. The total run time was 10 min at a flow rate of 0.5 mL/min, maintained at 40 °C, with a sample injection volume of 5.00 µL. Tandem mass spectrometry was performed using electrospray ionization (ESI) with Agilent Jet Stream technology in the positive mode. The analytes were measured with a gas temperature of 300 °C, a gas flow of 5 L/min, a nebulizer pressure of 45 psi, a sheath gas heater temperature of 375 °C, a sheath gas flow of 12 L/min, a capillary voltage of 3500 V, and a nozzle voltage of 500 V. The mass spectrometer operated in multiple reaction monitoring (MRM) mode, detecting transitions at m/z 315.2→193.1 and 315.1→123.0 for CBD. Additionally, the transition at 318.2→196.1 was detected for CBD-D3.

### 2.6. Sample Preparation

The sample extraction protocol was adapted from previous work [24]. One mL of acetonitrile (ACN) containing 1% formic acid (FA) and 10 μg mL− CBD-D3 was added to 200 μL of plasma in a 1.5 mL clear polypropylene tube and vortexed for 10 s. Following sonication of the mixture for 3 min, the samples were vortexed for 10 s and then centrifuged at 10,000 rpm for 5 min. All extraction steps, except drying, were conducted at room temperature. After centrifugation, 1 mL of the supernatant was transferred to a clean glass tube, and the solvent was evaporated using a SpeedVac at 60 °C. The dried samples were reconstituted in 200 μL of mobile phase (40% A and 60% B), followed by vortexing and sonication for 10 s and 3 min, respectively. The solution was transferred to a centrifuge tube and spun again at 10,000 rpm for 5 min. The resulting clean supernatant was collected and injected into the chromatographic system.

### 2.7. Pharmacokinetic Evaluation

In this study, the pharmacokinetic parameters were assessed through non-compartmental analysis (NCA). The individual pharmacokinetic parameters were estimated using the Phoenix WinNonlin 6.4 software. Pharmacokinetic analysis software was used to process the analytical data as a time versus plasma concentration comparison. The following parameters were examined: HL_Lambda_z (T_1/2_); terminal half-life: Cmax_D (Cmax); the maximum observed concentration divided by the dose: AUCINF_D_obs (AUC_0-inf_); AUC from the dosing time extrapolated to infinity, based on the last observed concentration, divided by dose: Vz_F_obs (Vz); the volume of the distribution associated with the terminal phase divided by F (bioavailability): Cl_F_obs (CL); clearance over F (based on the observed Clast): MRTINF_obs (MRT_inf_); the mean residence time extrapolated to infinity for a substance administered by extravascular dosing using the observed Clast: Lambda_z (Ke); the first-order rate constant associated with the terminal (log-linear) portion of the curve, estimated by the linear regression of time vs. the log concentration. The dose-normalized Cmax and AUC parameters were computed to enable the evaluation of the dose proportionality between the two groups.

### 2.8. Statistical Analysis

Descriptive and inferential statistical analyses (where applicable) were computed using Microsoft Excel and GraphPad Prism version 9.5.1 (733) for Windows (GraphPad Software; Boston, MA, USA). This included all outcome calculation data of the measure of central tendency (average; mean), variability (standard error mean), and figures. Statistical inference analysis determined significance at a 2-sided *p*-value < 0.05. Group comparisons were conducted using Mann–Whitney U tests. A comprehensive comparison of hematological and blood chemistry profiles was performed, analyzing the means and standard error of the means (SEMs) of the results. The obtained data were compared between three different time points using repeated measures ANOVA, with probability values less than 0.05 considered statistically significant.

## 3. Results

The present investigation examines the pharmacokinetics of CBD in mixed-breed canines. Animals in the OG cohort were administered CBD-infused oil at a dosage of 5 mg/kg, while those in the TG cohort received a single treat containing 50 mg of CBD. Pertinent pharmacokinetic parameters were derived using non-compartmental analysis for each dosage formulation. A summary of the PK parameter estimates is presented in Table 1.

A modified LC-MS/MS (MRM) protocol was conducted for the quantitative analysis of CBD in canine plasma specimens. The six-point calibration curve for various CBD concentrations ranged from 0.1 to 100 ng/mL, with an R^2^ value exceeding 0.9900. The method’s lower limit of quantification (LLOQ) was established at 0.1 ng/mL, demonstrating a reproducibility of 9.8% and an accuracy of 102.43%. This internally validated analytical approach enabled the precise measurement of CBD in dog plasma samples, including those with low analyte concentrations.

**Figure 1:**
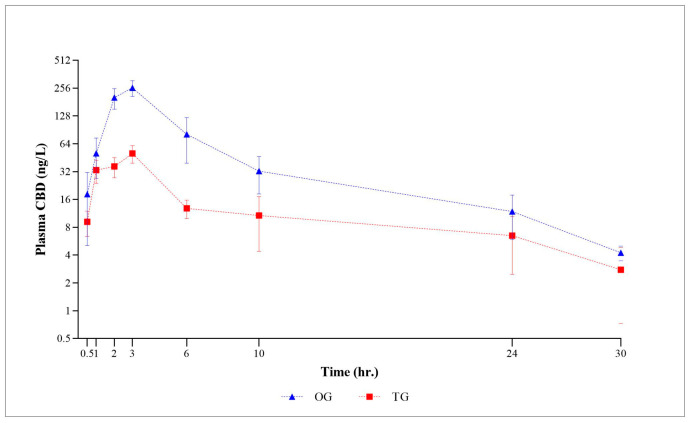
Graphical representation of semi-logarithmic scale of CBD plasma levels (mean ± SEM) after single oral dose administration in OG and TG groups.

The logarithmic graph in Figure 1 displays the mean plasma CBD concentrations (±SEM) for all time points in both CBD form groups. As depicted in Figure 1, the plasma CBD concentration, when plotted against the observation time points, demonstrates a consistent behavioral profile. Subsequently, the plasma concentration gradually decreased until 30 h post-administration. At the final 48 h time point, CBD was no longer detectable in the plasma. Following the administration of a single oral CBD oil-based and treat form, the plasma CBD concentrations exhibited considerable variability in each subject, ranging from 0.62 to 463.35 ng/mL and 1.02 to 105.76 ng/mL, with the calculated mean plasma concentrations of 82.29 ng/mL and 20.24 ng/mL, respectively. Regardless of the dosage form, the mean plasma concentration of the oil formulation was consistently higher at each time point compared to that of the treat formulation.

The pharmacokinetic analysis revealed substantial variations between the CBD-infused oil group (OG) and the CBD-mixed as a treat group (TG) under the investigated condition. Data in the table indicated that there are statistically significant differences between the OG and TG groups for Cmax_D, AUCINF_D_obs, and Cl_F_obs, with the TG group showing a lower Cmax_D and AUCINF_D_obs but higher clearance compared to the OG group. Other parameters did not show significant differences between the two groups. Notable findings include a statistically significant decline in a dose-normalized maximum plasma concentration within the TG (*p* = 0.0011), indicative of altered bioavailability or absorption kinetics. Relative bioavailability in the TG was markedly reduced, with values of 46.35% and 36.46% for AUC_D and Cmax_D, respectively. Furthermore, the TG exhibited a reduced dose-normalized area under the curve (*p* = 0.0499), pointing to a decrease in overall drug exposure.

The apparent clearance was observed to be elevated in the TG (*p* = 0.0499), potentially indicating accelerated drug elimination. With respect to the Cmax_D value, the calculated inter-individual variation for OG and TG was relatively similar at 39.91% and 41.84%, respectively. In addition, the TG showed a higher calculated inter-individual variation for AUCinf-D (95.97%) compared to the OG (69.23%), indicating greater variability in drug exposure among individuals in the TG. The OG reached the Tmax faster than the TG (2.38 ± 0.26 h vs. 3.63 ± 1.07 h), although this difference was not statistically significant. The result showed no statistically significant variations in half-life, time to maximum concentration, volume of distribution, or mean residence time among the groups. 

In the course of the study, the OG group displayed average levels ranging from 21 to 244 ng/mL, whereas the TG group demonstrated levels between 3 and 43 ng/mL, as detailed in Table 2. The concentrations in the OG group increased from day 1 to day 16, with a slight further increase by day 30, thus not following a straightforward linear pattern. Similarly, the TG group showed an increase over time, albeit this was less pronounced than the OG group, with a general upward trend observed until day 30. It was observed that the concentration of CBD in circulation generally increased over time but experienced a rapid decline following the cessation of administration.

During this study, we observed the health status and blood profiles of the studied dogs (Table S1). The TG group started with slightly higher values in several parameters. Overall, the data suggest that the CBD treatments had varying effects on the hematological and biochemical profiles of the dogs. The RBC, hemoglobin, and hematocrit levels increased over time in both groups but remained within the normal range, although some significant differences between times were observed in the OG group.

The MCHC levels increased from day 0 to day 15 in both groups. By day 30, the MCHC levels decreased slightly in both groups but remained within the normal range. The eosinophil counts decreased significantly from day 0 to day 15, with a further decrease by day 30. The protein and albumin levels increased over time in both groups, with the TG group showing higher levels. Although certain blood parameters, including the MCHC, eosinophil, plasma protein (biuret), and albumin, showed a significant difference between times of exposure, all of the parameters remained in the normal range. The OG group had a slower but more steady improvement, while the TG group showed peaks followed by stabilization. The biochemical parameters for liver and kidney function showed some fluctuations in enzyme levels. No clinically significant adverse effects were observed in the hematological or biochemical parameters across all CBD forms. Furthermore, no dogs experienced adverse effects or exhibited signs of abnormality throughout the experimental period.

## 4. Discussion

The findings of the study on canine plasma following a single administration of 5 mg/kg CBD-infused oil (OG) and 50 mg of CBD-mixed as a treat (TG) over 30 days provided valuable insights into the pharmacokinetics of CBD in dogs. The pharmacokinetic profile of CBD in canines has been extensively investigated, with numerous studies elucidating its behavior in this species [10]. Typically, it is characterized by low oral bioavailability [15,17] and moderate distribution [6,25].

The Cmax and AUC values observed in this study are consistent with previous research on CBD pharmacokinetics in dogs, contingent upon the formulation and dosage [5,11,14,17]. Furthermore, the Cmax in the TG group was comparable to the absorption levels reported in earlier studies involving soft chews [11]. The higher Cmax and AUC in the OG group suggest that this formulation may enhance CBD absorption compared to the TG group. This observation aligns with the findings that dogs administered CBD in an oil-based formulation exhibited a higher Cmax and AUC compared to those given a capsule formulation [17]. These results corroborate the existing literature, indicating that the formulation type significantly influences CBD absorption in canines. For instance, a study comparing CBD-infused oil to microencapsulated CBD oil beads found that the microencapsulated beads resulted in lower Cmax and AUC values, suggesting reduced bioavailability compared to the oil formulation [10]. Another investigation evaluated a soft chew containing a 50:50 mixture of CBD and cannabidiolic acid (CBDA) administered at 2 mg/kg, achieving a Cmax of approximately 300 ng/mL for CBD, which was higher than the ~100 ng/mL observed with an oil-based formulation at the same dosage [11]. This suggests that certain edible formulations may enhance CBD absorption. Additionally, a study assessing various CBD preparations found that the liquid formulations (oil-based, nanoemulsion-based, and water-soluble) provided higher bioavailability and faster absorption compared to the semi-solid form [14]. The relative bioavailability of CBD in the TG compared to the OG group indicated a reduced overall exposure and peak concentration in the treat formulation. The current findings confirmed a trend for oil formulations to result in a faster Tmax than treat or soft-gel formulations [11,14]. Furthermore, solid dosage forms tend to have reduced bioavailability, highlighting the challenge of formulating CBD effectively in these forms. The treat formulation likely presented a different matrix that may have hindered the dissolution and absorption of CBD, leading to reduced bioavailability. The treat’s ingredients and excipient interaction with CBD may reduce releasing and/or absorption in the gastrointestinal tract. Collectively, the treat formulation in this study required nearly a two times higher dose than the oil formulation to achieve comparable AUC values, suggesting reduced bioavailability due to food matrix interactions. Despite differing doses, a dose-normalized Cmax and AUC were analyzed to assess relative absorption. The longer Tmax for the snack form indicates a delayed release, which may influence dosing regimens in clinical use.

In contrast, soft-gel capsules showed higher maximum serum concentrations for CBD and its metabolites compared to the oil formulations [13]. These studies underscore the impact of formulation on CBD pharmacokinetics in dogs, with oil-based and certain edible forms demonstrating varying degrees of bioavailability, highlighting the necessity of considering the formulation type when evaluating CBD absorption and therapeutic efficacy in canines.

It is widely recognized that plasma concentrations of CBD in canines are influenced by the route of administration, dosage, formulation characteristics, and individual factors. It is noteworthy that the oral transmucosal administration of 1 mg/kg CBD resulted in a mean Cmax comparable to that observed with oral administration [26]. This finding implies that the administration route may not significantly affect plasma concentrations at relatively low doses.

The present data corroborate the accumulation of CBD in plasma over time, consistent with previous studies that indicate that repeated administration results in elevated plasma levels, thereby supporting the plasma accumulation hypothesis [9,12]. However, upon cessation, CBD levels in the current study declined rapidly, suggesting the presence of active clearance mechanisms. Vaughn and colleagues demonstrated that CBD exhibits linear pharmacokinetics with repeated administration, where plasma concentrations increase proportionally with the dose [12]. As the dose increases, the volume of distribution for CBD may also increase due to its lipophilicity and tendency to accumulate in fatty tissues. This can lead to greater systemic exposure over time with repeated dosing [5].

The observed plasma accumulation and extended elimination half-life suggest that CBD may reach steady-state levels with chronic administration, reinforcing its predictable pharmacokinetic profile in canines. This was further substantiated by a study indicating that chronic administration of CBD to dogs resulted in dose-proportional accumulation over 36 weeks, with an increased half-life, total exposure, mean residence time, and plasma peak level [9].

CBD generally has a short half-life, lasting a few hours after a single administration. The current findings showed that the half-life was similar between the OG and TG groups, with no significant difference, suggesting comparable elimination rates of CBD in both groups. This study confirmed previous findings that the half-life of CBD in dogs ranges from approximately 6 to 10 h [12,14,15,17]. This may support the hypothesis that CBD pharmacokinetics are largely independent of minor formulation changes, at least in terms of elimination.

The present study utilized refined rice bran oil enriched with γ-oryzanol as a carrier for CBD, distinguishing it from prior canine pharmacokinetic studies that predominantly employed different oily vehicles, including sunflower lecithin, medium-chain triglyceride (MCT) oil, and sesame oil [5,11,13,17]. MCT oils are widely used in canine formulations; this may be due to their rapid absorption via portal circulation [27], though their lack of endogenous antioxidants may limit long-term stability compared to plant-derived oils like rice bran oil.

To our knowledge, this represents the first systematic evaluation of rice bran oil for CBD delivery in veterinary applications. Its balanced fatty acid profile (approximately 20% saturated, 40% monounsaturated, and 35% polyunsaturated) coupled with native antioxidant compounds (notably γ-oryzanol at 1.5–2.5% *w*/*w*) may offer distinct advantages for chronic administration. γ-Oryzanol not only stabilizes the formulation against oxidative degradation [28] but may also exert synergistic anti-inflammatory effects with CBD, as demonstrated in murine models [29]. These pharmacodynamic interactions warrant further investigation in canine systems.

Additionally, dosing frequency alteration may be necessary to maintain therapeutic effects, particularly for conditions requiring long-term management. However, inter-study variability highlights the impact of multiple factors on pharmacokinetic outcomes. Taken together, CBD pharmacokinetics are known to be highly variable in dogs, both within and between studies. Factors such as breed, individual metabolic differences, dosage, formulation, feeding status, and duration of administration can all influence plasma concentrations [5,14,25,30].

While CBD is generally well-tolerated in canines, its effects on blood parameter profiles have yielded mixed results, particularly concerning the liver enzymes and other biochemical markers [5,15,19,31]. The present study identified alterations in specific hematological parameters. Notably, within the erythrocyte profile, the OG group exhibited a significant increase in red blood cell (RBC) count and hemoglobin levels, along with a near-significant rise in hematocrit, suggesting mild erythropoietic stimulation. CBD interacts with the endocannabinoid system, which plays a role in hematopoiesis. Some studies suggested that endocannabinoids can stimulate hematopoiesis [32]. Controversially, an in vitro study suggested that CBD may affect the integrity of erythrocytes, potentially leading to the formation of hemolytic vesicles [33].

Regarding the leukocyte profile, a significant decrease in eosinophil levels was observed in both groups, indicating potential anti-inflammatory or anti-allergic effects. In a murine asthma model, administration of CBD-X resulted in a significant reduction in eosinophil infiltration within lung tissues, suggesting its potential to mitigate eosinophil-driven inflammation [34]. This finding implies that CBD may exert anti-inflammatory effects by modulating immune cell activity, particularly by reducing eosinophil counts. However, the OG group demonstrated reduced lymphocytes, possibly reflecting immunomodulatory activity. For platelet and coagulation markers, the mean platelet volume (MPV) and platelet large cell ratio (P-LCR) exhibited significant reductions in both groups, suggesting CBD may influence platelet activation or turnover.

In relation to the biochemical safety profiles observed in this study, minor fluctuations were noted in the levels of alanine aminotransferase (ALT) and aspartate aminotransferase (AST). Specifically, the AST levels exhibited a slight increase in the OG group, while the ALT levels decreased in the TG group. Alkaline phosphatase (ALP) demonstrated a significant reduction in the TG group, possibly due to formulation-specific effects. However, the levels of ALT, AST, and ALP remained within normal parameters, thereby confirming the absence of hepatotoxicity concerning liver function. Certain studies reported that most hematological and serum chemistry variables remained within normal ranges for both the CBD-treated and control groups; some dogs showed elevated alkaline phosphatase levels in response to CBD treatment [19,20]. This indicates that individual dogs may respond differently to CBD supplementation, possibly due to variations in metabolism or sensitivity. Additionally, blood urea nitrogen (BUN) and creatinine (CRE) exhibited no significant variations, indicating no renal impairment. However, with respect to protein metabolism, there was a significant increase in total protein and albumin levels in both the OG and TG groups, suggesting enhanced protein synthesis or retention. A recent investigation into the safety and pharmacokinetics of a CBD-rich hemp extract in rats over a 90-day period found that female rats developed significant hyperalbuminemia following CBD administration, whereas male rats did not exhibit this increase [35]. This outcome contrasts with findings from another study, which identified significant differences in albumin levels between treatment groups at weeks 18 and 26, with mean concentrations approximately 2 g/L lower in CBD-treated dogs. Nonetheless, these values remained within the normal reference range [19]. A non-significant positive association was observed in several parameters, with no obvious clinical incidence of adverse events in this current study.

Collectively, the OG group demonstrated erythropoietic and anti-inflammatory effects without adverse hepatic or renal impact. Additionally, the TG group exhibited mild immunomodulatory effects (eosinophil reduction) but no significant hematological alterations. Both formulations were well-tolerated over 30 days, supporting their safety in canine use. However, while CBD is generally well-tolerated in dogs, elevated liver enzymes (particularly ALP) are the most notable adverse effect, warranting periodic monitoring.

Certain limitations of this study merit consideration, particularly its small sample size consisting of crossbreed dogs. This may have resulted in the limited statistical power, thereby increasing the risk of not identifying clinically significant differences in the pharmacokinetic and blood parameters between groups. Moreover, this study could not thoroughly assess inter-individual variability in CBD metabolism, which may be influenced by factors such as CYP450 polymorphisms or body composition, thus constraining the generalizability of the results. It should be noted that inter-individual variation in drug responsiveness is not limited to CBD but is a common phenomenon in laboratory animals. Löscher (2024) emphasizes that despite standardized breeding and husbandry, phenotypic diversity exists in laboratory mice and rats, affecting various characteristics of animal disease models, including drug responsiveness. This variation can lead to misinterpretations when data are averaged within experimental groups, as divergent responses may be concealed [36]. The use of different doses between formulations in this study may introduce confounding factors in direct pharmacokinetic comparisons, despite dose-normalization adjustments. Furthermore, the food matrix of the snack formulation (e.g., fat content) may have independently influenced drug absorption, thereby introducing variabilities that are not fully accounted for in the analysis. Additionally, the assumption of dose-linear pharmacokinetics may not be valid if saturation effects occur at higher doses. These factors should be considered when evaluating the therapeutic implications under real-world dosing conditions. Future research involving larger and more diverse cohorts is necessary to validate these preliminary results and to refine the dosing recommendations.

## 5. Conclusions

Conclusively, this comprehensive 30-day study on cannabidiol’s pharmacokinetics and health effects in dogs, comparing oil and treat forms of administration, has the potential to significantly advance our understanding of this compound in veterinary medicine. Moreover, this study provides preliminary evidence that CBD supplementation may modulate erythropoiesis and immune responses in dogs, warranting further long-term studies to establish safe dosing thresholds and assess the cumulative effects and its therapeutic potential.

## Figures and Tables

**Table 1:** PK parameters (mean ± SEM) of CBD following a single oral dose administration in the OG and TG groups, where *p*-values < 0.05. The dose-normalized Cmax and AUC parameters are presented.

Parameter	Unit	OG (*n* = 8)				TG (*n* = 8)				*p*-Value
HL_Lambda_z (T_1/2_)	h									
Mean ± SEM		9.66	±	1.52		8.52	±	1.15		0.7984
Range (Median)		4.87	-	15.1	(8.5)	1.8		12.75	(8.95)	
95% CI		6.68	-	12.63		6.25		10.78		
Tmax	h									
Mean ± SEM		2.38	±	0.26		3.63	±	1.07		0.5793
Range (Median)		1	-	3	(2.5)	1		10	(3)	
95% CI		1.85	-	2.89		1.53		5.71		
Cmax_D	kg*ng/mL/mg									
Mean ± SEM		58.40	±	8.24		21.29	±	3.15		0.0011
Range (Median)		21.15		92.66	(55.7)	8.2		37.12	(19.06)	
95% CI		42.24		74.55		15.11		27.46		
AUCINF_D_obs	h*kg*ng/mL/mg									
Mean ± SEM		305.85	±	74.86		141.75	±	48.10		0.0499
Range (Median)		70.11		752.7	(268)	59.93		475.2	(96.49)	
95% CI		159.1		452.6		47.48		236		
Vz_F_obs	L/kg									
Mean ± SEM		88.11	±	34.25		115.18	±	22.40		0.2345
Range (Median)		9.35		295.4	(43)	30.76		227.7	(123.7)	
95% CI		20.97		155.2		71.27		159		
Cl_F_obs	L/h/kg									
Mean ± SEM		5.12	±	1.45		9.99	±	1.45		0.0499
Range (Median)		1.32		14.26	(3.74)	2.1		16.68	(10.37)	
95% CI		2.26		7.96		7.15		12.82		
MRTINF_obs	h									
Mean ± SEM		8.54	±	1.19		9.83	±	1.78		0.6454
Range (Median)		5.23		15.93	(7.69)	5.81		21.54	(9.11)	
95% CI		6.2		10.84		6.33		13.32		
Lambda_z (Ke)	1/h									
Mean ± SEM		0.09	±	0.01		0.11	±	0.04		0.7984
Range (Median)		0.04		0.14	(0.08)	0.05		0.38	(0.07)	
95% CI		0.05		0.11		0.03		0.19		
Relative AUC_D (TG/OG)		46.35								
Relative Cmax_D (TG/OG)		36.46								

**Table 2:** Display the plasma CBD concentration (mean ± SEM) for the OG and TG groups (ng/mL) as collected on the pre-determination day during the study.

Day	OG (ng/mL)		TG (ng/mL)	
	Mean	SEM	Mean	SEM
0	ND		ND	
1	202.14	50.92	36.41	8.97
2	119.14	23.85	21.66	4.21
16	237.92	47.93	37.35	4.81
30	244.90	41.06	43.09	8.48
31	21.58	4.34	3.89	1.24

## Data Availability

Data are contained within the article.

## Supplementary Materials

The following supporting information can be downloaded at: https://www.mdpi.com/article/10.3390/ani15101470/s1, Table S1: Hematology and blood chemistry test: OG and TG group of dogs that received CBD for 30 days.

## References

[1] Jurga M., Jurga A., Jurga K., Kaźmierczak B., Kuśmierczyk K., Chabowski M. (2024). Cannabis-Based Phytocannabinoids: Overview, Mechanism of Action, Therapeutic Application, Production, and Affecting Environmental Factors. Int. J. Mol. Sci..

[2] Silver R.J. (2019). The Endocannabinoid System of Animals. Animals.

[3] Kosukwatthana P., Rungsuriyawiboon O., Rattanasrisomporn J., Kimram K., Tansakul N. (2024). Cytotoxicity and Immunomodulatory Effects of Cannabidiol on Canine PBMCs: A Study in LPS-Stimulated and Epileptic Dogs. Animals.

[4] Tihăuan B.M., Onisei T., Slootweg W., Gună D., Iliescu C., Chifiriuc M.C. (2025). Cannabidiol—A Friend or a Foe?. Eur. J. Pharm. Sci..

[5] Gamble L.J., Boesch J.M., Frye C.W., Schwark W.S., Mann S., Wolfe L., Brown H., Berthelsen E.S., Wakshlag J.J. (2018). Pharmacokinetics, Safety, and Clinical Efficacy of Cannabidiol Treatment in Osteoarthritic Dogs. Front. Vet. Sci..

[6] McGrath S., Bartner L.R., Rao S., Packer R.A., Gustafson D.L. (2019). Randomized Blinded Controlled Clinical Trial to Assess the Effect of Oral Cannabidiol Administration in Addition to Conventional Antiepileptic Treatment on Seizure Frequency in Dogs with Intractable Idiopathic Epilepsy. J. Am. Vet. Med. Assoc..

[7] Alvarenga I.C., Panickar K.S., Hess H., Mcgrath S. (2025). Scientific Validation of Cannabidiol for Management of Dog and Cat Diseases CBD: Cannabidiol. Annu. Rev. Anim. Biosci..

[8] Nerapusee O., Soontornvipart K., Pettong T., Phongsuchat N., Lunsucheep D., Patikorn C., Vimolmangkang S., Anantachoti P. (2023). Thai Veterinarians’ Perceptions of Cannabidiol Products for Dogs with Osteoarthritis: A Qualitative Interview Study. J. Small Anim. Pract..

[9] Corsato Alvarenga I., MacQuiddy B., Duerr F., Elam L.H., McGrath S. (2023). Assessment of Cannabidiol Use in Pets According to a National Survey in the USA. J. Small Anim. Pract..

[10] Di Salvo A., Conti M.B., della Rocca G. (2023). Pharmacokinetics, Efficacy, and Safety of Cannabidiol in Dogs: An Update of Current Knowledge. Front. Vet. Sci..

[11] Wakshlag J.J., Schwark W.S., Deabold K.A., Talsma B.N., Cital S., Lyubimov A., Iqbal A., Zakharov A. (2020). Pharmacokinetics of Cannabidiol, Cannabidiolic Acid, Δ9-Tetrahydrocannabinol, Tetrahydrocannabinolic Acid and Related Metabolites in Canine Serum After Dosing with Three Oral Forms of Hemp Extract. Front. Vet. Sci..

[12] Vaughn D.M., Paulionis L.J., Kulpa J.E. (2021). Randomized, Placebo-Controlled, 28-Day Safety and Pharmacokinetics Evaluation of Repeated Oral Cannabidiol Administration in Healthy Dogs. Am. J. Vet. Res..

[13] Tittle D., Wakshlag J., Schwark W., Lyubimov A., Zakharov A., Gomez B. (2022). Twenty-Four Hour and One-Week Steady State Pharmacokinetics of Cannabinoids in Two Formulations of Cannabidiol and Cannabidiolic Acid Rich Hemp in Dogs. Med. Res. Arch..

[14] Limsuwan S., Phonsatta N., Panya A., Asasutjarit R., Tansakul N. (2024). Pharmacokinetics Behavior of Four Cannabidiol Preparations Following Single Oral Administration in Dogs. Front. Vet. Sci..

[15] Bartner L.R., McGrath S., Rao S., Hyatt L.K., Wittenburg L.A. (2018). Pharmacokinetics of Cannabidiol Administered by 3 Delivery methods at 2 Different Dosages to Healthy Dogs. Can. J. Vet. Res..

[16] Polidoro D., Temmerman R., Devreese M., Charalambous M., Van Ham L., Cornelis I., Broeckx B.J.G., Mandigers P.J.J., Fischer A., Storch J. (2022). Pharmacokinetics of Cannabidiol Following Intranasal, Intrarectal, and Oral Administration in Healthy Dogs. Front. Vet. Sci..

[17] Deabold K.A., Schwark W.S., Wolf L., Wakshlag J.J. (2019). Single-Dose Pharmacokinetics and Preliminary Safety Assessment with Use of CBD-Rich Hemp Nutraceutical in Healthy Dogs and Cats. Animals.

[18] Pacher P., Kogan N.M., Mechoulam R. (2020). Beyond THC and Endocannabinoids. Annu. Rev. Pharmacol. Toxicol..

[19] Bradley S., Young S., Bakke A.M., Holcombe L., Waller D., Hunt A., Pinfold K., Watson P., Logan D. (2022). Long-Term Daily Feeding of Cannabidiol Is Well-Tolerated by Healthy Dogs. Front. Vet. Sci..

[20] Morris E.M., Kitts-Morgan S.E., Spangler D.M., Gebert J., Vanzant E.S., McLeod K.R., Harmon D.L. (2021). Feeding Cannabidiol (CBD)-Containing Treats Did Not Affect Canine Daily Voluntary Activity. Front. Vet. Sci..

[21] Cunningham J.B., McCrum-Gardner E. (2007). Power, Effect and Sample Size Using GPower: Practical issues for Researchers and Members of Research Ethics Committees. Evid. Based Midwifery.

[22] McRae G., Melanson J.E. (2020). Quantitative Determination and Validation of 17 Cannabinoids in Cannabis and Hemp Using Liquid Chromatography-Tandem Mass Spectrometry. Anal. Bioanal. Chem..

[23] ICH Guideline (2022). M10 Bioanalytical Method Validation and Study Sample Analysis Guidance for Industry. https://www.fda.gov/media/162903/download.

[24] Jamwal R., Topletz A.R., Ramratnam B., Akhlaghi F. (2017). Ultra-High Performance Liquid Chromatography Tandem Mass-Spectrometry for Simple and Simultaneous Quantification of Cannabinoids HHS Public Access. J. Chromatogr. B Anal. Technol. Biomed. Life Sci..

[25] Vaughn D., Kulpa J., Paulionis L. (2020). Preliminary Investigation of the Safety of Escalating Cannabinoid Doses in Healthy Dogs. Front. Vet. Sci..

[26] Della Rocca G., Paoletti F., Conti M.B., Galarini R., Chiaradia E., Sforna M., Dall’Aglio C., Polisca A., Di Salvo A. (2023). Pharmacokinetics of Cannabidiol Following Single Oral and Oral Transmucosal Administration in Dogs. Front. Vet. Sci..

[27] Roopashree P.G., Shetty S.S., Suchetha Kumari N. (2021). Effect of Medium Chain Fatty Acid in Human Health and Disease. J. Funct. Foods.

[28] Juliano C., Cossu M., Alamanni M.C., Piu L. (2005). Antioxidant Activity of Gamma-Oryzanol: Mechanism of Action and Its Effect on Oxidative Stability of Pharmaceutical Oils. Int. J. Pharm..

[29] Rao Y.P.C., Sugasini D., Lokesh B.R. (2016). Dietary Gamma Oryzanol Plays a Significant Role in the Anti-Inflammatory Activity of Rice Bran Oil by Decreasing pro-Inflammatory Mediators Secreted by Peritoneal Macrophages of Rats. Biochem. Biophys. Res. Commun..

[30] Kogan L., Hellyer P., Downing R. (2020). The Use of Cannabidiol-Rich Hemp Oil Extract to Treat Canine Osteoarthritis-Related Pain: A Pilot Study. AHVMA J..

[31] Corsato Alvarenga l., Wilson K.M., McGrath S. (2024). Tolerability of Long-Term Cannabidiol Supplementation to Healthy Adult Dogs. J. Vet. Intern. Med..

[32] Randall M.D. (2007). Endocannabinoids and the Haematological System. Br. J. Pharmacol..

[33] Gómez C.T., Borda N., Moscovicz F., Fernandez F., Lazarowski A., Auzmendi J. (2024). In Vitro Effect of Cannabidiol on Red Blood Cells: Implication in Long-Lasting Pathology Treatment. Curr. Pharm. Des..

[34] Aswad M., Pechkovsky A., Ghanayiem N., Hamza H., Dotan Y., Louria-Hayon I. (2024). High-CBD Extract (CBD-X) in Asthma Management: Reducing Th2-Driven Cytokine Secretion and Neutrophil/Eosinophil Activity. Pharmaceuticals.

[35] Dehner J., Polanska H.H., Petrlakova K., Zeljkovic S.C., Beres T., Hendrych M., Storch J., Tarkowski P., Masarik M., Babula P. (2025). Safety Assessment on CBD-Rich Hemp Extract in Sub-Chronic Cross-Sex Study with Rats. Toxicol. Appl. Pharmacol..

[36] Löscher W. (2024). Of Mice and Men: The Inter-individual Variability of the Brain’s Response to Drugs. eNeuro.

