# Supplementary material for: Pharmacokinetic Characteristics of a Single Cannabidiol Dose in Oil and Treat Forms and Health Impacts After 30 Days of Administration in Dogs
Source: Animals (Basel). 2025 May 19;15(10):1470. doi: 10.3390/ani15101470 (PMC12108255; doi:10.3390/ani15101470)
Supplement: Supplementary file 1 [file animals-15-01470-s001.zip › animals-3606049-supplementary.pdf]

Table S1: Hematology and blood chemistry test: OG and TG group of dogs that received CBD for 30 days.

Hematology and Blood chemistry test: OG and TG group of dogs received CBD for 30 days.

The obtained data were subjected to compare between three different time points by repeated measure ANOVA, with probability values less than 0.05 considered statistically significant.

**Parameter**		**CBD-oil (OG group)**			**CBD-treat (TG group)**		
(Abbreviation; normal range)	Date	Mean	SD	P value	Mean	SD	P value
Red blood cell	Day0	6.31	0.58	0.004	6.8	1.07	0.254
RBC (4.95 - 7.87 10^ 6/uL)	Day15	6.74	0.54		7.5	1.17	
	Day30	6.95	0.29		6.87	1.17	
Hemoglobin	Day0	14.59	1.14	0.002	15	2.41	0.277
HGB (11.9-18.9 g/dL)	Day15	15.78	1.28		16.63	2.05	
	Day30	16.36	0.67		15.65	2.65	
Hematocrit	Day0	44.76	3.43	0.019	46.18	6.98	0.654
HCT (35-57 %)	Day15	46.09	3.82		48.75	6.04	
	Day30	48.58	1.40		46.75	7.48	
Mean corepuscular volume	Day0	71.1	2.75	0.001	67.96	3.41	<0.001
MCV (66-77fL)	Day15	68.41	2.10		65.3	2.67	
	Day30	69.93	1.81		68.28	2.73	
Mean corepuscular hemoglobin	Day0	23.2	1.24	0.317	22.09	1.07	0.007
MCH (21-26.2pg)	Day15	23.43	1.03		22.28	1.09	
	Day30	23.55	0.76		22.81	0.98	
Mean corepuscular hemoglobin concentration	Day0	32.58	0.68	<0.001	32.46	0.69	0.003
MCHC(21-26.2g/dl)	Day15	34.28	1.07		34.13	1.00	
	Day30	33.65	0.55		33.45	0.80	
Platelet count	Day0	257.13	32.74	0.004	282.25	36.88	0.078
PLT (211-621 10^ 3/uL)	Day15	246.75	39.19		251.75	46.43	
	Day30	283	43.74		234.75	44.76	
Red cell Distribution Width-SD	Day0	33.36	2.43	0.249	31.98	1.91	0.711
RDW-SD (39-46fL)	Day15	31.84	3.05		31.31	3.94	
	Day30	32.4	1.73		32.31	3.32	
Red cell Distribution Width-CV	Day0	12.65	0.37	0.605	13.45	1.40	0.351
RDW-CV (11.6-14.6%)	Day15	13.06	1.25		14.3	2.91	
	Day30	12.98	1.22		13.63	1.74	
Platelet Distribution Width	Day0	13.9	5.27	0.625	14.6	7.83	0.538
PDW (9.1-19.4fL)	Day15	13.84	5.45		12.68	6.67	

	Day30	13.76	2.35		13.9	5.35	
Mean Platelet Volume MPV (8.7-13.2fL)	Day0	11.13	0.78	0.023	11.21	0.79	0.062
	Day15	11.13	0.77		10.48	0.74	
	Day30	10.73	0.88		10.66	0.61	
Platelet Large Cell Ratio P-LCR (%)	Day0	36.95	6.67	0.003	37.69	6.03	0.077
	Day15	35.86	7.32		31.89	6.74	
	Day30	32.39	7.94		32.2	5.26	
Plateletcrit PCT (0.14-0.46%)	Day0	0.16	0.08	0.906	0.11	0.06	0.074
	Day15	0.18	0.04		0.13	0.08	
	Day30	0.17	0.06		0.17	0.07	
Nucleated red blood cell NRBC (0-1%)	Day0	0.01	0.04	0.293	0.05	0.05	0.918
	Day15	0.01	0.04		0.04	0.08	
	Day30	0.05	0.08		0.05	0.05	
White blood cell WBC (5-14.1 10^ 3/uL)	Day0	10.84	3.06	0.110	11.84	2.90	0.082
	Day15	9.24	2.57		9.2	2.19	
	Day30	9.81	2.14		10.57	2.11	
Neutrophils NEUT (2.9-12 10^3/uL)	Day0	6.05	2.05	0.046	7.35	2.77	0.114
	Day15	5.68	2.02		4.97	1.53	
	Day30	7.08	1.81		7.22	2.55	
Lymphocytes LYMPH (0.4-2.9 10^ 3/uL)	Day0	2.56	0.54	0.010	3.17	0.92	0.173
	Day15	2.31	0.67		3.14	0.88	
	Day30	1.8	0.55		2.56	1.15	
Monocytes MONO (0.1-1.4 10^ 3/uL)	Day0	0.5	0.20	0.253	0.5	0.13	0.317
	Day15	0.4	0.17		0.41	0.13	
	Day30	0.39	0.14		0.51	0.15	
Eosinophils EO (0-1.3 10^ 3/uL)	Day0	1.72	0.97	0.001	1.41	0.53	<0.001
	Day15	0.84	0.27		0.47	0.18	
	Day30	0.53	0.18		0.47	0.17	
Basophils BASO (0-0.14 10^ 3/uL)	Day0	0.02	0.01	0.084	0.03	0.01	0.044
	Day15	0.02	0.01		0.02	0.01	
	Day30	0.01	0.01		0.02	0.01	
Blood urea nitrogen BUN (8-28 mg%)	Day0	18.25	4.77	0.086	20.5	4.14	0.520
	Day15	18	3.51		20.5	3.96	
	Day30	15	2.83		22.25	2.87	
Creatinine CRE (0.50-1.70 mg%)	Day0	1.2	0.16	0.036	1.24	0.19	0.044
	Day15	1.09	0.11		1.05	0.16	

	Day30	1.08	0.13		1.07	0.14	
Alanine aminotransferase ALT (10-109 U/L)	Day0	27.25	5.63	0.121	30.13	9.60	0.015
	Day15	20.75	8.91		23	6.63	
	Day30	26.13	10.26		26.13	8.74	
Aspartate aminotransferase AST (13-15)	Day0	13.5	1.20	0.032	13.5	1.60	0.824
	Day15	14.75	1.16		13.38	1.69	
	Day30	15	2.33		13.75	1.98	
Alkaline phosphatase ALP (8-76 U/L)	Day0	45.13	16.09	0.414	38	14.55	0.004
	Day15	42.13	21.50		31.75	16.19	
	Day30	54.38	43.12		41.88	18.89	
PROTEIN biuret PROTEIN biuret (5.4-7.5 g/dl)	Day0	5.91	0.27	0.002	6.09	0.44	0.011
	Day15	6.35	0.30		6.69	0.44	
	Day30	6.39	0.27		6.86	0.54	
ALBUMIN ALBUMIN (2.3-3.1g/dl)	Day0	3.09	0.15	0.000	3.09	0.14	0.001
	Day15	3.25	0.19		3.48	0.23	
	Day30	3.46	0.25		3.5	0.25	
AMYLASE AMYLASE (226-1,063 U/L)	Day0	170.75	20.63	0.014	164.63	23.48	0.002
	Day15	220.63	54.37		211.38	28.48	
	Day30	212	62.47		205.63	36.97	
